# Comparative study of stress characteristics in surrounding bone during insertion of dental implants of three different thread designs: A three‐dimensional dynamic finite element study

**DOI:** 10.1002/cre2.152

**Published:** 2018-12-26

**Authors:** Chaiwat Udomsawat, Pimduen Rungsiyakull, Chaiy Rungsiyakull, Pathawee Khongkhunthian

**Affiliations:** ^1^ Center of Excellence for Dental Implantology, Faculty of Dentistry Chiang Mai University Thailand; ^2^ Department of Prosthodontics, Faculty of Dentistry Chiang Mai University Thailand; ^3^ Department of Mechanical Engineering, Faculty of Engineer Chiang Mai University Thailand

**Keywords:** dynamic finite element, implant design, implants insertion, stress distribution

## Abstract

The objective of this study is to evaluate the stress distribution characteristics around three different dental implant designs during insertion into bone, using dynamic finite element stress analysis. Dental implant placement was simulated using finite element models. Three implants with different thread and body designs (Model 1: root form implant with three different thread shapes; Model 2: tapered implant with a double‐lead thread; and Model 3: conical tapered implant with a constant buttress thread) were assigned to insert into prepared bone cavity models until completely placed. Stress and strain distributions were descriptively analyzed. The von Mises stresses within the surrounding bone were measured. At the first 4‐mm depth of implant insertion, maximum stress within cortical bone for Model 3 (175 MPa) was less than the other models (180 MPa each). Stress values and concentration area were increasing whereas insertion depth increased. At full implant insertion depth, maximum stress level in Model 1 (35 MPa) within the cancellous bone was slightly greater than in Models 2 (30 MPa) and 3 (25 MPa), respectively. Generally, for all simulations, the highest stress value and the location of the stress concentration area were mostly in cortical bone. However, the stress distribution patterns during the insertion process were different between the models depending on the different designs geometry that contacted the surrounding bone. Different implant designs affect different stress generation patterns during implant insertion. A range of stress magnitude, generated in the surrounding bone, may influence bone healing around dental implants and final implant stability.

**List of abbreviations**
FEA: Finite Element Analysis
MPa: Megapascal

## 1 INTRODUCTION

Various studies have shown that in current dental practice, dental implants have been accepted as a standard treatment for edentulous patients and have shown a long‐term success rate of as high as 97% after 10 years of function (Fugazzotto, 2005). High performance in treatment and successful applications has been claimed for the various materials and designs of each implant system. The critical function of a dental implant is transferring occlusal force to the surrounding bone at the bone‐implant interface (Abuhussein, Pagni, Rebaudi, & Wang, 2010).

The greater the bone‐implant contact surface, the more force distribute to surrounding bone. There are three primary types of loads generated at the bone‐implant interface: compressive, tensile, and shear forces. Studies have shown that compressive forces lead to increased bone density and strength. Tensile and shear forces have been shown to result in weaker bones with shear forces being the least beneficial (Havaldar, Pilli, & Putti, 2014; Oftadeh, Perez‐Viloria, Villa‐Camacho, Vaziri, & Nazarian, 2015). Factor that has had an impact on the stress in the surrounding bone is the macroscopic implant design.

The macroscopic parts of the implant fixture, including the body and thread geometry, directly contact the bone during the implant insertion process (Vandeweghe, Cosyn, Thevissen, Teerlinck, & De Bruyn, 2012). At first, threaded cylindrical dental implants with parallel walls were shown to be successful over long periods of time. However, more recent implant configurations have begun to incorporate a tapered form. Advantages of the tapered, or root form, implant over the cylindrical form include less space required in the apical region (allowing for placement in narrow spaces or in narrow regions with labial or lingual concavities), better primary stability for immediate placement into alveolar sockets, and better distribution of the compressive forces (Garber, Salama, & Salama, 2001).

Another important part of the dental implant geometry is the thread. Threads are effective at increasing the initial contact with the surrounding bone through converting rotary motion into linear motion, contributing to primary stability. However, they exhibit differences in how they transmit loads to the adjacent bone. Consequently, it has been reported that the face angle of the thread changes the direction of the force at the bone‐implant interface (Misch, 2007). The amounts of shear force generated by the different thread shapes are increased as the thread face angle increases. In previous studies on thread configuration and force distribution in a static condition, implants with either squared or buttress threads reported that the axial load of these implants were mostly dissipated through a compressive force (Hansson & Werke, 2003; Misch, 2007), whereas V‐shaped and reverse buttress‐threaded implants transmitted the axial force through a combination of compressive, tensile, and shear forces (Desai, Desai, Katti, & Karthikeyan, 2012).

Apart from the thread geometric design, some manufacturers have introduced multiple‐threaded implants, which have two or more threads running parallel, one to the other. These multiple‐threaded implants allow for faster insertion, but according to one finite element analysis (FEA) study, the most favorable configuration in terms of implant stability appeared to be the single‐lead threaded implant, followed by the double‐lead threaded implant. The triple‐lead threaded implant was found to be the least stable (Abuhussein et al., 2010).

The concept of the implantation process is to prepare the bone site with a specific dimensional cavity using sequenced drill bits. Then, the implant is finally inserted manually into the bone cavity in a revolving manner, using a torque ratchet or mechanically, using a surgical micromotor. Part of the dental implant geometry contact, compress, or penetrate the surrounding bone. The bone surrounding the implant responds to contact the implant surface through a complex biomechanical process, which affects the osseointegration. The appropriate stress magnitude generated in the insertion process minimizes bone damage and promotes bone healing. The response of the bone in terms of resorption or healing is directly related to the stress within the bone, in accordance with Wolff's theory (Brand, 2010; Mavčič & Antolič, 2012).

Several studies have reported the relationship between the implant geometric design and force distribution in a static condition (Abuhussein et al., 2010; Herekar, Patil, Mulani, Sethi, & Padhye, 2014; Oswal, Amasi, Oswal, & Bhagat, 2016). However, more research is needed to evaluate the force distribution or stress generation in the surrounding bone in a dynamic rotary insertion condition.

The purpose of this study was to compare the stress in the surrounding bone produced during implant insertion by different dental implant designs, using dynamic FEA.

## 2 MATERIAL AND METHODS

### 2.1 Finite element modelling

**Figure 1:**
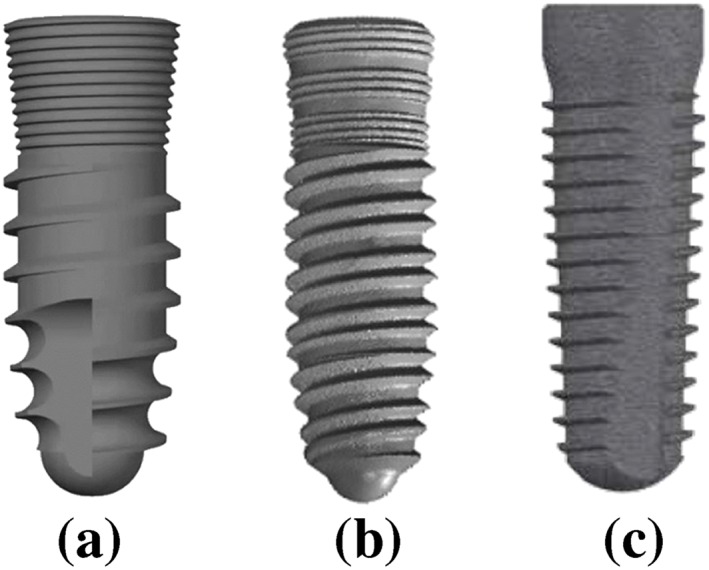
Implants used in implantation simulation. (a) Model 1: root form implant with three different thread shapes, (b) Model 2: tapered implant with a double‐lead thread, (c) Model 3: Conical tapered implant with a constant buttress thread

Three different implant fixtures with a unique form and thread shape were used as reference to model three dental implants, using a SolidWorks (DS SolidWorks Corp., Waltham, MA, USA) 3D‐CAD program (Figure 1). The first was a PW plus® dental implant (PW Plus, Nakhon Pathom, Thailand), which has a unique tapered root form with three different thread shapes on the body (Model 1). From the top of the implant fixture, the microthread was located at the collar area, the reverse buttress thread was located in the middle of the body, and a condensed thread was located at the apical third of the fixture. The thread of the implant began with a condensed thread (near the apex of the implant), which gradually increased in thickness along the body of the implant (Tirachaimongkol, Pothacharoen, Reichart, & Khongkhunthian, 2016). The implant had a diameter of 3.75 mm and length of 12 mm with a three‐thread‐design tapered screw.

The second design was a tapered NDI implant (PW Plus, Nakhon Pathom, Thailand) with a double‐lead V‐shape thread (Model 2). The double‐lead thread ran parallel along the implant body from the apical third and finished at the microthread zone. The implant had a diameter of 3.8 mm and was 12 mm in length.

The third dental implant design was a CONELOG® SCREW‐LINE dental implant (Camlog® Biotechnologies, Wimsheim, Germany). The implant was designed with a 3° conical, tapered body geometry, and outside had a self‐tapping thread pattern with a buttress shape (Model 3). The implant had a diameter of 3.8 mm and was 11 mm in length.

**Figure 2:**
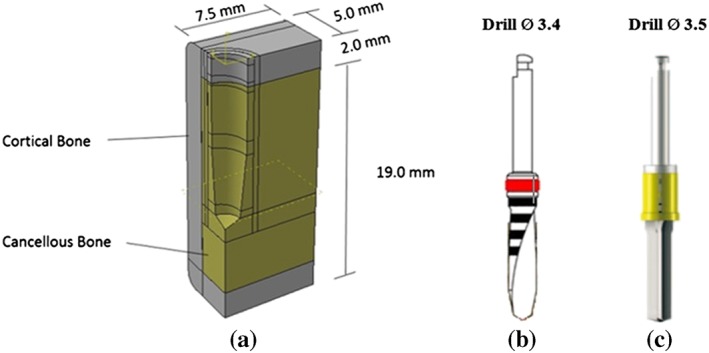
(a) The dimensions of the bone segment model, (b) Final drill bit of Models 1 and 2, (c) Final drill bit of Model 3

Each dental implant was inserted into a prepared cavity in a 90° mandibular segment, (Figure 2a), in order to reduce computational requirements. The bone was modeled as a cancellous core covered by a 2.0‐mm cortical layer. The mesial and distal section planes were not covered by cortical bone. The prepared cavity dimensions in the bone model were constructed following the shape of the final drill of each implant system. The same prepared cavity dimensions were used for the PW plus® and NDI dental implants (12 mm in depth and 3.4 mm in diameter from the top then tapered to 2.4 mm beginning at 5 mm before the apex), which conformed to the implant body geometry. On the other hand, a form drill, which had a diameter of 3.5 mm, length of 11 mm, and tapered 3° along the length to the apex, was used to prepare the cavity for the CONELOG® SCREW‐LINE implants. The dimensions of the bone segment are indicated in Figure 2.

**Figure 3:**
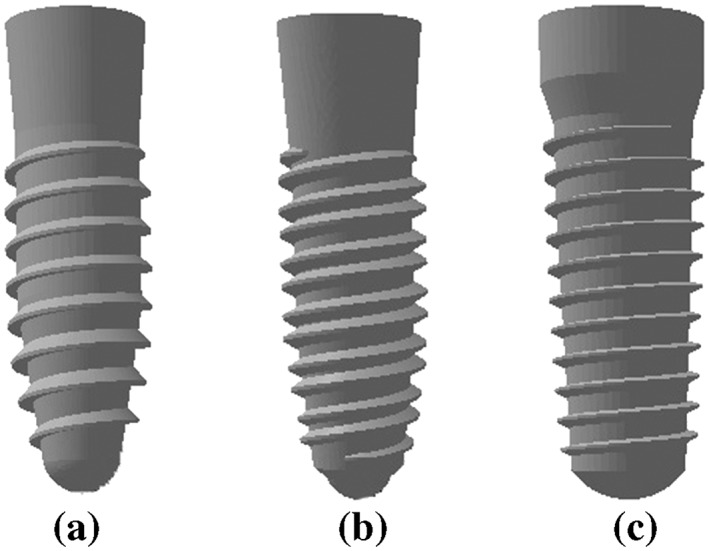
Three different implants after model simplification

To maintain a successful compromise between the computational time and the accurate representation of the implant insertion, the implant models were simplified by reducing some of the geometry. This was conducted for two reasons: First, some of the geometry created severe displacement of the bone elements, and second, the geometric zone that had no direct contact with the bone was not affected by stress during the insertion process. Therefore, the microthread zone of Models 1 and 2 were removed, as well as the cutting faces of Models 1 and 3 (Figure 3).

### 2.2 Materials properties

All materials used in the models were considered to be isotropic, homogeneous, and linearly elastic. The realistic material behavior of the cancellous and cortical bone were simulated through the definition of elastic and plastic properties, as listed in Table 1. The elastic plastic behavior of the bone was defined by Young's modulus, Poisson's ratio, density, yield stress, and plastic strain. Cancellous bone properties with Young's modulus of 1.37 GPa and a Poisson's ratio of 0.31 were assigned to the posterior mandible. The Young's moduli and Poisson's ratios for the cortical bone were 14 GPa and 0.30, respectively.

**Table 1:** Material properties

Materials	Young's modulus *E* (10^3^ N/mm^2^)	Poisson ratio	Density, *ρ* (10^−7^ kg/mm3)
Cortical bone	9	0.3	18
Cancellous bone	0.7	0.31	5.3
Titanium	102	0.35	45

### 2.3 Simulation techniques

The simulation of the implant insertion process in this study was conducted using the ABAQUS 6.5.4 Finite Element software (SIMULIA Corporation, Providence, RI, USA). The implicit solver of ABAQUS was efficient for solving the nonlinear problem of rotating the implant into the bone. Each implant was meshed with 6586–7558 C3D8R (Continuum, 3‐D, 8‐node, reduced integration) hexahedral elements. Both cortical and cancellous bone were meshed with 72,698–85,755 C3D8R hexahedral elements (the total number of bone elements was 72,698–85,755), providing a solution of equivalent accuracy at less cost. The small elements were meshed in the region around the drilling cavity, which was 0.1 mm in size.

A symmetry condition was applied on the plane where the thread‐forming process occurred, with the normal pointing in the directions of the x and y axes. The “encastre” feature was applied to outer surface of the bone model to prevent any rigid body motion.

**Figure 4:**
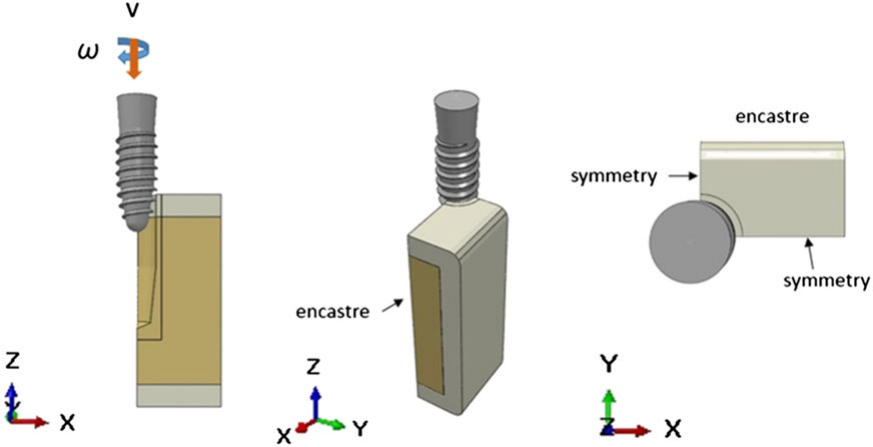
Boundary condition

To simulate the insertion process, the implant body was allowed only to rotate and move downwards in one axis. A continuous, constant, angular velocity in the range of 50–60 rpm was applied at the reference point of the implant. A constant vertical linear velocity was calculated in relation to the number of pitches and the total insertion time period of 18 s (Figure 4).

The contact between the surfaces was defined by the surface‐to‐surface discretization method. In addition, the contact interaction properties were required to be defined for the contact pair. All of the implant surfaces were assigned to rigid body constraint, defined as the master surface, and all of the surrounding bone elements were assigned to a slave surface. The constraint of the contact between the two types of bone was assigned to tie the degree of freedom constraint. The tangential properties at the bone‐implant interface was defined using a friction coefficient, and the normal properties were defined as a hard contact. The hard contact was implemented to ensure that the master nodes had complete contact with the slave surfaces, therefore, not allowing the transfer of any tensile stresses across the interface.

### 2.4 Stress measurements

**Figure 5:**
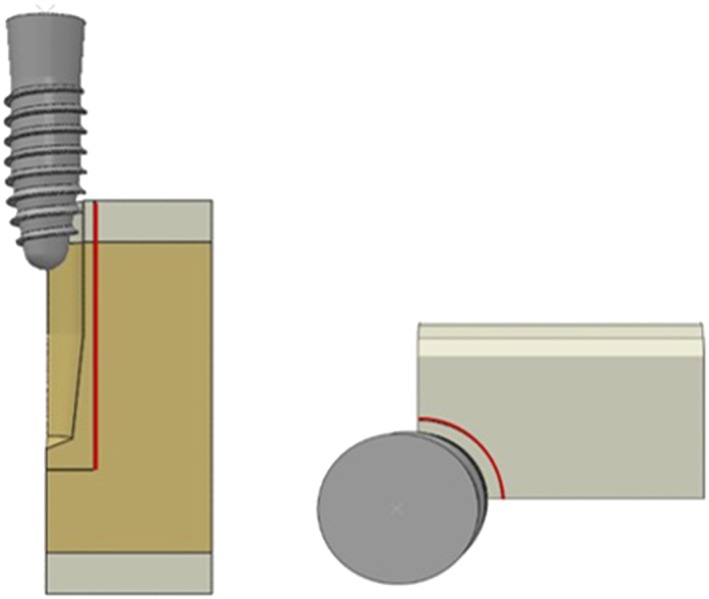
Stress measurement line

The von Mises stresses were recorded along a predetermined line every 1 mm on the vertical axis both in the cortical bone and the cancellous bone, beginning when the implant made contact with the bone and continuing until fully completing the implantation. The vertical line was 15.0 mm in length and 0.5 mm away from the bone‐implant contact surface. The stress distribution magnitude was presented as a stress profile plot, showing the stress value in elements through which the predetermined line passed. Moreover, color coding, that was available in the software, was used to show the areas in the model with different stress magnitudes. The colored bar at the side of the analysis showed the maximum stress component (red) and minimum stress component (blue) generated when the analysis was conducted. The highest von Mises stress value and pattern of the stress distribution were compared in all implants under similar insertion conditions (Figure 5).

## 3 RESULTS

### 3.1 Model 1

#### 3.1.1 Cortical bone

**Figure 6:**
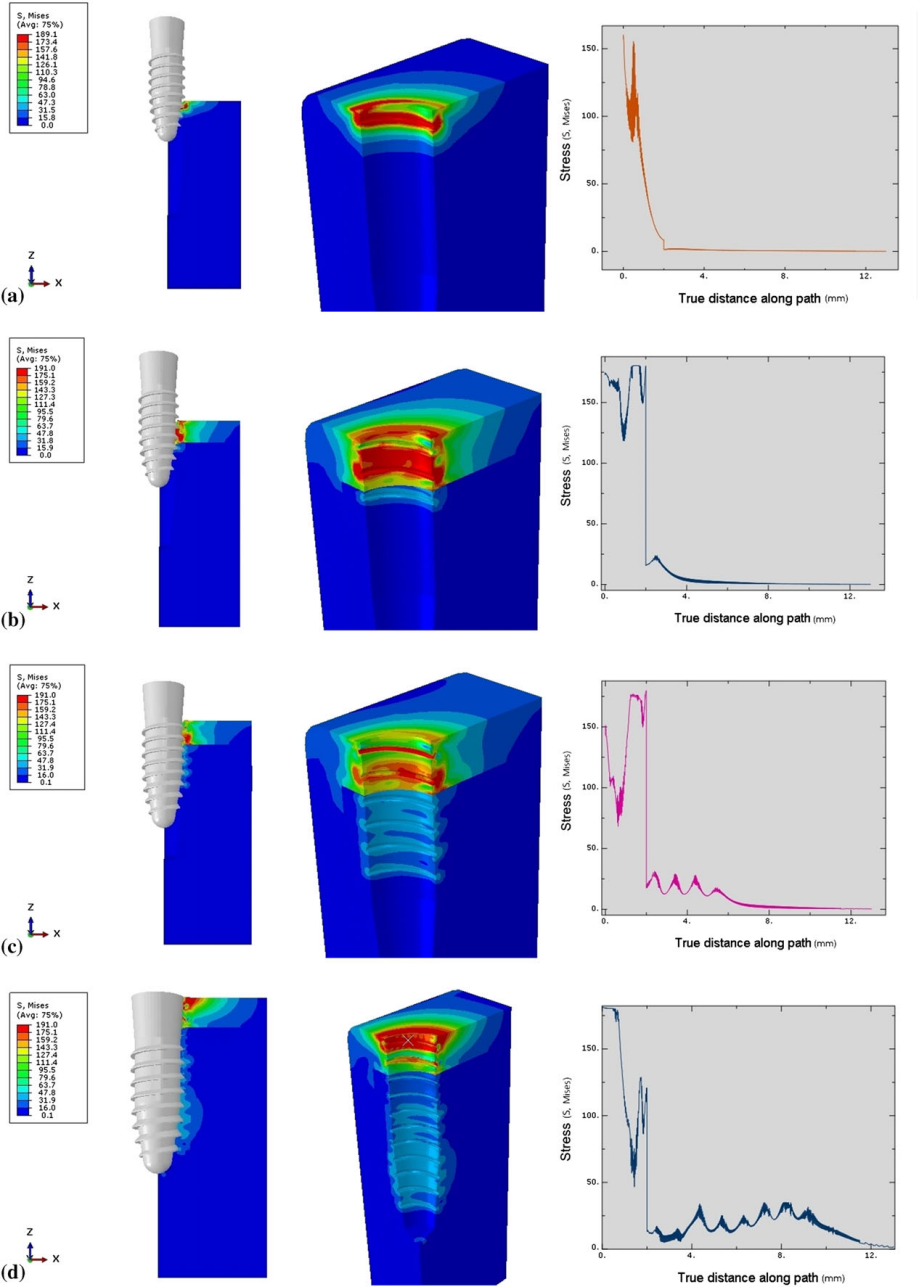
Von Mises stresses characteristic in surrounding bone in Model 1. (a) Stress profile at 3‐mm insertion depth, (b) Stress profile at 6‐mm insertion depth, (c) Stress profile at 9‐mm insertion depth, (d) Stress profile at 12‐mm insertion depth

At the beginning of the insertion process, the first contact between the implant and the prepared bone cavity began at the third crest (rotation of the thread) of the implant thread as the implant body penetrated 3 mm into the bone (Figure 6a). The thread‐forming process compressed the surrounding bone, progressively increasing the stress values during the rotation of the implant. The stress values reached a peak (180.00 MPa) when the implant moved into the cylindrical zone (6 mm depth; Figure 6b). Then, the stress was distributed into a more even pattern and gradually decreased after the implant revolved to the fifth rotation as a result of the thread moving along the formed groove in the bone, without any change of thread geometry. At the final stage of insertion, when the implant was inserted 10 mm in depth, the stress concentration gradually increased until the insertion process was complete because the diameter of the implant body was wider than that of the cavity (Figure 6d). Therefore, the stress profile in the cortical bone margin was created by the compression of the implant collar.

#### 3.1.2 Cancellous bone

**Figure 7:**
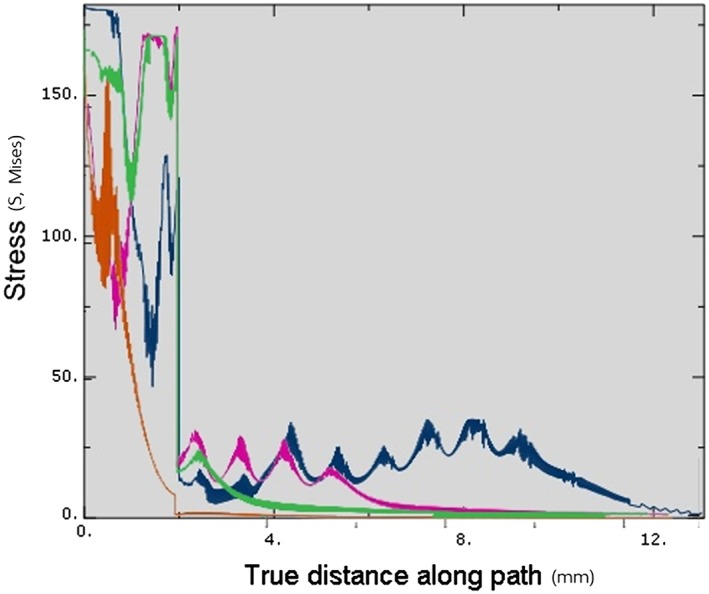
Total von Mises stresses in Model 1 at each insertion depth. Brown—Stress value at 3‐mm insertion depth, Green—Stress value at 6‐mm insertion depth, Pink—Stress value at 9‐mm insertion depth, Blue—Stress value at 12‐mm insertion depth

Similar to the cortical bone, the stress in the cancellous bone also increased when the thread‐forming process began. However, the stress values in the cancellous bone were much less than in the cortical bone due to the material properties of the bone (Young's modulus and density). The stress in the surrounding cancellous bone was shown at the first contact of the implant surface and was distributed evenly as the thread moved along the groove (12.0–30.0 MPa; Figure 6b,c). Upon complete insertion, the stress increased slightly (25.0–35.0 MPa) 7.0–9.0 mm from the bone surface, as a result of increased contact between the thread surface and the bone (Figure 6d; Figure 7).

### 3.2 Model 2

#### 3.2.1 Cortical bone

**Figure 8:**
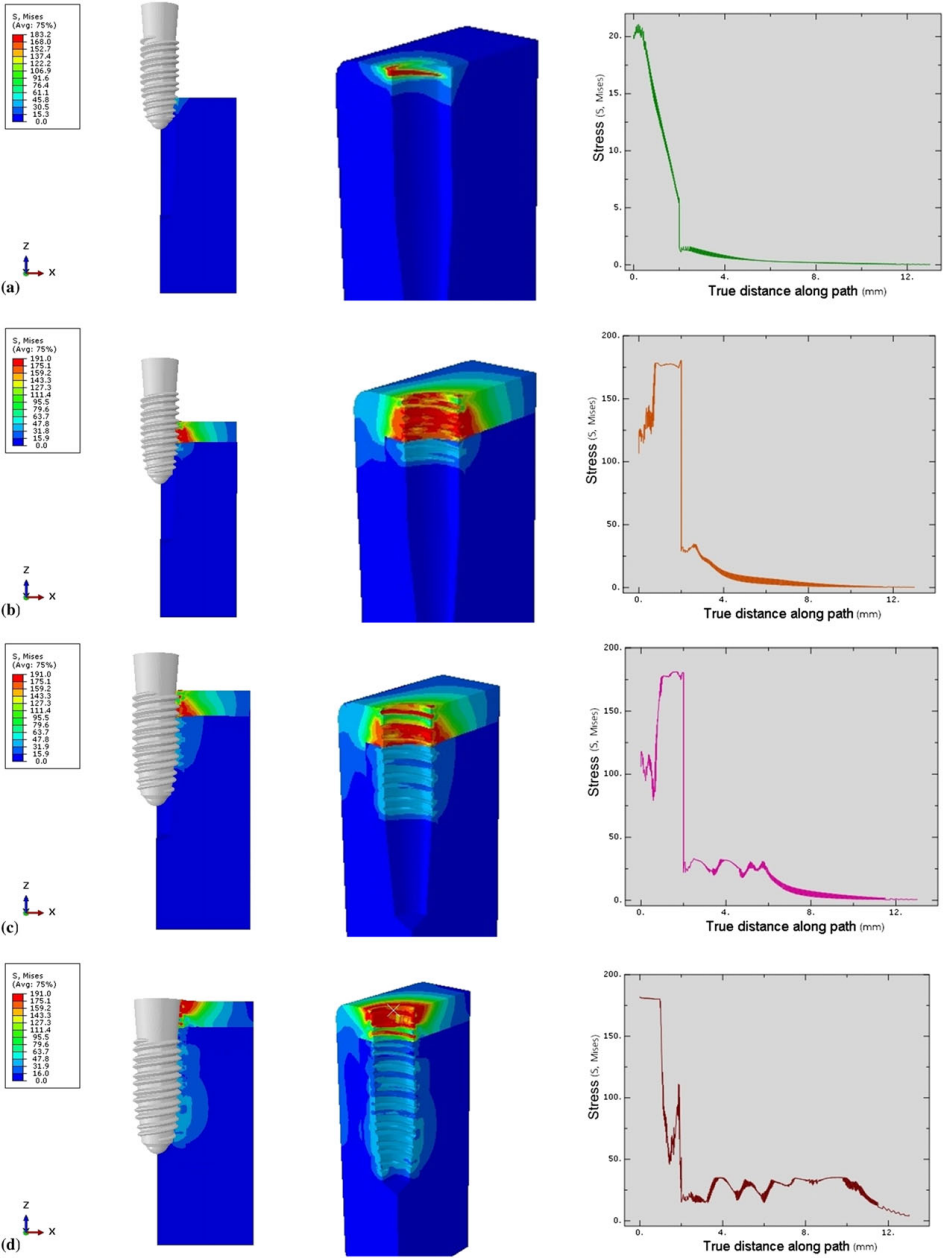
Von Mises stresses characteristic in surrounding bone in Model 2. (a) Stress profile at 3‐mm insertion depth, (b) Stress profile at 6‐mm insertion depth, (c) Stress profile at 9‐mm insertion depth, (d) Stress profile at 12‐mm insertion depth

The implant did not have any contact with the bone until it penetrated to a depth of 3 mm from the top surface of the bone (Figure 8a). For the implant in Model 2, which had two start threads, the thread‐forming process began with the second start thread followed by the first start thread. At an insertion depth of 3–5 mm, the stress value increased in the bone as a result of compression by both threads. The peak stress magnitude (180 MPa) was present 0.4 mm from the top surface of the bone, where the third crest of both threads made contact. At an insertion depth of 5–10 mm (Figure 8b,c), the stress (90–180 MPa) gradually decreased due to the constancy of the geometry of the thread as it moved along the formed groove in the bone. At the end of the insertion process, the widest part of the implant (the neck) compressed the margin of the bone, producing a stress magnitude of 170 MPa (Figure 8d).

#### 3.2.2 Cancellous bone

**Figure 9:**
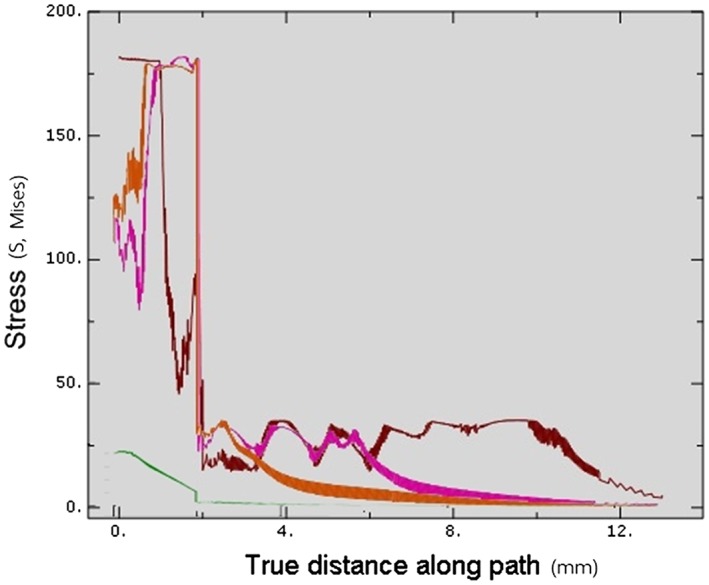
Total von Mises stresses in Model 2 at each insertion depth. Green—Stress value at 3‐mm insertion depth, Orange—Stress value at 6‐mm insertion depth, Pink—Stress value at 9‐mm insertion depth, Dark red—Stress value at 12‐mm insertion depth

The first contact of the implant in the cancellous bone was different from that in the cortical bone. The first contact began with the first start thread at 0.4 mm from the cancellous border, followed by the second start thread at 1 mm below the cancellous border (Figure 8a). The stress level gradually increased simultaneously with the thread‐forming process. The stress (25‐30 MPa) was distributed more evenly as the thread moved along the formed groove (Figure 8c). Upon complete insertion, the stress magnitude (30 MPa) in the bone surrounding the apical 1/3 of the implant was distributed over a larger area than elsewhere, due to the increased implant surface contact with the bone in that area (Figures 8d and 9.

### 3.3 Model 3

#### 3.3.1 Cortical bone

**Figure 10:**
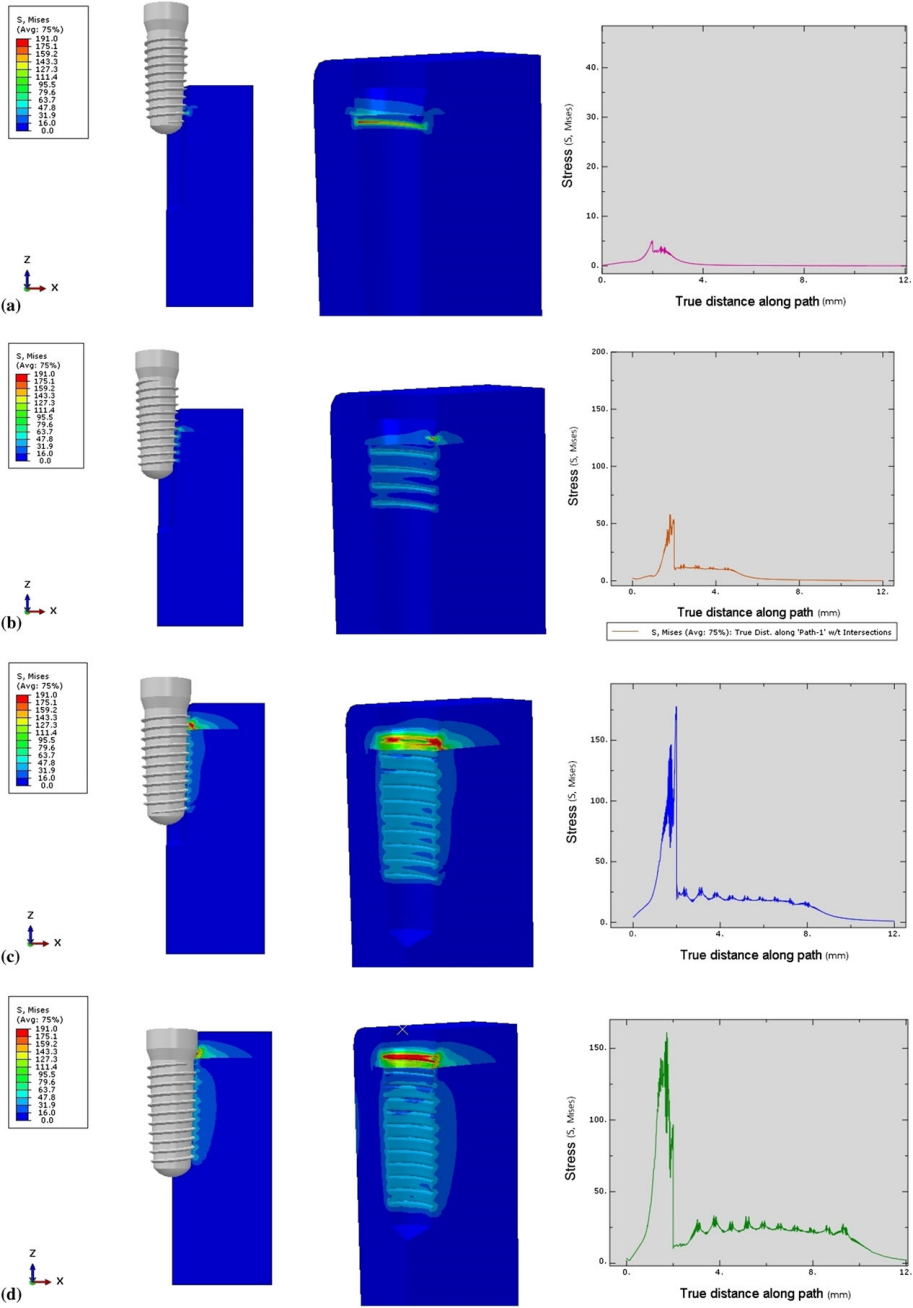
Von Mises stresses characteristic in surrounding bone in Model 3. (a) Stress profile at 3‐mm insertion depth, (b) Stress profile at 6‐mm insertion depth, (c) Stress profile at 9‐mm insertion depth, (d) Stress profile at 11‐mm insertion depth

The stress was generated by the first contact of the third crest of the thread when the implant penetrated 4 mm into the bone (Figure 10a). After that, the thread‐forming process progressively increased the stress level. The peak stress magnitude (175 MPa) was found at the 10th crest of the thread (Figure 10d). Then, the stress gradually decreased (60–175 MPa) until the implant rotated to the end of the thread. Some residual stress was present at the bone next to the formed groove. Upon complete insertion, only the surface of the collar compressed the cortical bone (Figure 10d).

#### 3.3.2 Cancellous bone

**Figure 11:**
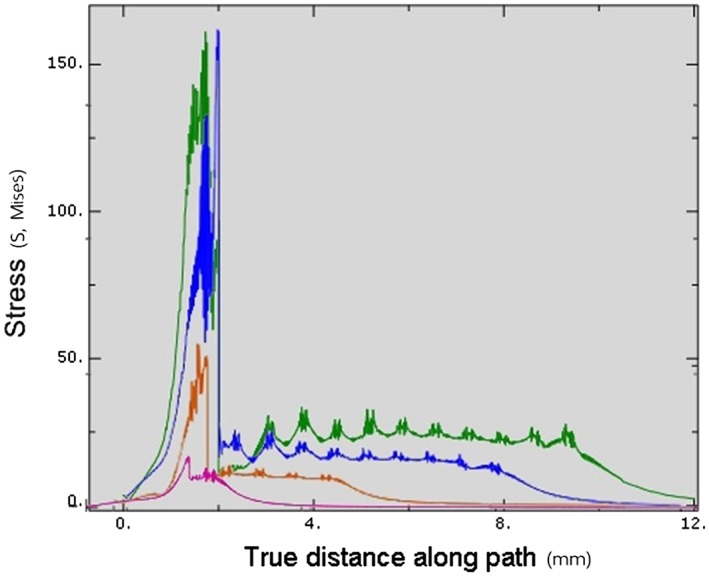
Total von Mises stresses in Model 3 at each insertion depth. Pink—Stress value at 3‐mm insertion depth, Orange—Stress value at 6‐mm insertion depth, Blue—Stress value at 9‐mm insertion depth, Green—Stress value at 11‐mm insertion depth

The first contact was begun at 0.4 mm from the cancellous border at the second crest of the thread (Figure 10a). The stress pattern was distributed more evenly in the cancellous bone than in the cortical bone as the thread moved and formed the groove until the implant was fully inserted (Figures 10d and 11.

### 3.4 Model comparison

**Figure 12:**
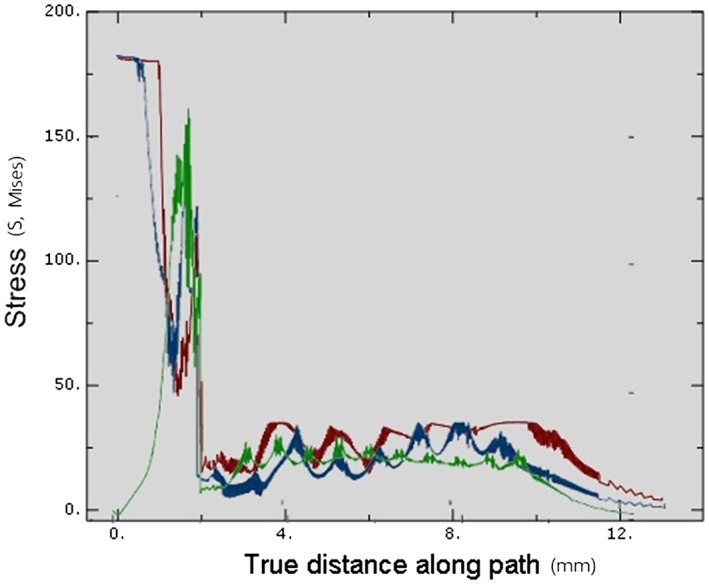
Von Mises stresses comparing in all models at full insertion depth. Blue—Stress value of Model 1, Dark red—Stress value of Model 2, Green—Stress value of Model 3

Upon complete insertion, the stress magnitude within the cortical bone in Model 3 (175 MPa) was less than in other models (180 MPa). Within the cancellous bone, the stress level in Model 1 (35 MPa) was slight higher than in Models 2 (30 MPa) and 3 (25 MPa). However, Model 2 showed a greater stress area than did Model 1, as there was a greater thread‐contact surface area in the apical region. Generally, for all simulations, the stress level in the cortical bone was significantly higher than in the cancellous bone (Figure 12, Table 2).

**Table 2:** Maximum von Mises stress in each model

Model	Maximum von Mises stress (MPa)	
	Cortical bone	Cancellous bone
Model 1	180	35
Model 2	180	30
Model 3	175	25

**Figure 13:**
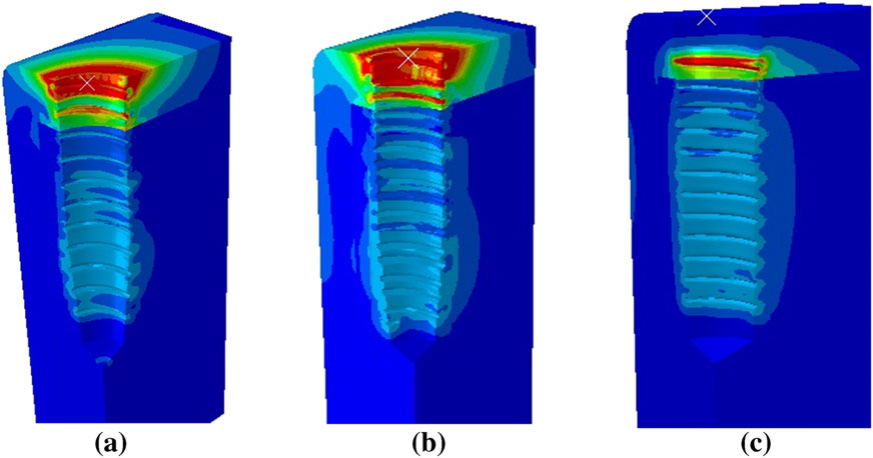
Stress distribution area in surrounding bone in each model

Upon complete insertion, the areas of greatest stress concentration in Models 1 and 2 were at the cortical bone surface (Figure 13), due to the compression of the bone by the implant collar and differences in diameter between the implant bodies and the prepared cavities. On the other hand, in Model 3, the stress at the cortical bone surface was intense in a smaller area than in the other models, with no stress showing at the bone surface. In the cancellous bone, the stress was distributed more evenly in Model 3 than in Models 1 and 2 (Figure 13).

## 4 DISCUSSION

For stress analysis problems involving complicated geometries, such as in bone and dental implant systems, it is very difficult to achieve an analytical solution. The finite element method has been used in such problems to analyze the stress distribution patterns in the bone‐implant interface for different implant designs (Geng, Tan, & Liu, 2001). Overloading stress in surrounding bone can cause resorption of the bone and failure of the implant. Therefore, investigation of stress field in surrounding bone is important (Esposito, Thomsen, Ericson, Sennerby, & Lekholm, 2000). In this study, the characteristics of stress distribution in the surrounding bone during implant insertion were investigated. The results demonstrated that the continuous increase in the dimension of the thread geometry and in the bone‐implant contact area increases stress magnitude in the surrounding bone.

### 4.1 Effect of the dental implant design on stress distribution during implantation

The functions of the implant thread are similar to those of a normal mechanical screw (Rüedi, Buckley, & Moran, 2018). The first function is to engage the surrounding bone, giving the screw stability, and the second is to change the rotational movement of the implant to a linear movement. When the implant is inserted, the thread gradually compresses the bone rotationally, creating a thread shape in the surrounding bone. The implant is rotated along the thread forming a groove until fully inserted. As the threads and body geometry of the implant increase in width whereas the implant is moving, stress is generated. The more abrupt the increase in the geometry, the more the thread surface penetrates or compresses the surrounding bone, producing additional stress. In Models 1 and 2, the thread began with a small thickness and gradually increased in size along the body. From this thread design, stress in the surrounding bone also increased as the insertion depth increased.

An implant thread design with a constant thread size (depth and width) and shape throughout the body would produce less stress in the surrounding bone than would one with a variable thread size, as was the case in Model 3.

According to Guan et al., different stress characteristics are produced by different sizes of implant surface contact areas (Guan, van Staden, Johnson, & Loo, 2011). Shamami et al. state that the loading direction has a significant effect on the maximum stress values and stress distribution patterns in the implant/bone system (Shamami, Karimi, Beigzadeh, Derakhshan, & Navidbakhsh, 2014). Their continuous dynamic study emphasized that the size and shape of the thread geometry contacting the bone was a major factor characterizing the stress profile in the surrounding bone.

### 4.2 The importance of the relationship between the final drill bit and prepared cavity

The dimension of the prepared cavity was created by the drilling process of the dental implant final drill bit. The shape of the final drill bit was designed to cut the bone into an implant‐conformed shape but not perfectly similar to the implant geometry. To use an appropriately sized final drill bit specific to the implant diameter and length is recommended for most implant systems (Ramasamy, Giri, Subramonian, & Narendrakumar, 2013).

The implantation process distributes complex loading forces in various directions to the surrounding bone (Shamami, Karimi, Beigzadeh, Derakhshan, & Navidbakhsh, 2014). The loading direction has a significant effect on the maximum stress values and stress distribution patterns in the implant/bone system. Their study showed that the stress profile in the surrounding bone of the prepared cavity is distributed more evenly than when the implant geometry slightly displaced the surrounding bone. An abrupt increase in the implant geometry creates stress to the bone. Pritchard stated that a prepared cavity that was much smaller than the implant geometry would receive increased stress as the implant moved (Pritchard, 1973). If the stress magnitude was beyond the bone yield point, the surrounding bone would be damaged. On the other hand, if the cavity was larger than the implant geometry, then there would be less engagement between the bone and thread, with a decreased effect on implant stability in the fully inserted position. Therefore, the diameter of the final drill is critical for the osteotomy because the bone next to this drill would have direct initial contact with the implant. The greater the geometrical differences between the implant and bone cavity, the greater the bone area that would be compressed, and the greater the stress generated. Duyck stated that the difference in size between the implant collar and the prepared cavity plays an important role in bone strains and stresses when an implant is inserted (Duyck et al., 2010). As seen in the implant collar areas in Models 1 and 2 (Figure 13a,b), the smaller the bone cavity, the greater the stress generated by the implant compression, leading to crestal bone loss. Therefore, the clinician should use the proper sized final drill matched to the implant size for each specific patient.

When implants were inserted into appropriately prepared bone sites matching the insertion characteristics of the three models**,** the implant body compressed the surrounding bone upon complete insertion. Compression of soft bone provides not only greater stability but also greater bone‐implant contact than if there is no compression, both of which would be a benefit during the initial bone‐loading process and also when bone density is poor (Giannasi et al., 2018)**.**

### 4.3 Effect of the bone component on stress distribution

The amount of stress is directly correlated to the force applied to the bone, for instance, through the loading of an oral implant (Abuhussein et al., 2010). But, the stress is also dependent on the mechanical properties of the bone (Brand, 2010; Mavčič & Antolič, 2012).

In this study, most of the maximum stresses were located next to the implant within the cortical bone rather than in the cancellous bone (Figure 13). This location was due to two material factors, the first of which was the elastic property of each bone type. Type II cortical bone properties were assigned with Young's modulus (*E)* of 9, which was higher than 0.7 in the cancellous bone (Burstein, Reilly, & Martens, 1976). The greater the modulus, the stiffer the material. Therefore, the cortical bone had less ability to deform than did the cancellous bone. The second factor was the material density, whereby the greater density of cortical bone generated greater stress than did cancellous bone because of their different Young's modulus (Clift, Fisher, & Watson, 1992). Consequently, the cortical bone needed greater force to be deformed than did cancellous bone. Moreover, the greater density and Young's modulus of cortical bone imply that the same amount of force can result in different amount of stress in bones with different properties.

Therefore, implant designers must be aware of not only biomechanical but also biomaterial problems, such as tissue response to different types of load transmission into the bone‐implant interface. The maximum stresses are located in surrounding bone around the implant collar, which should be both clinically and structurally strong enough to functionally maintain the interface. (Razaghi, Biglari, & Karimi, 2017).

### 4.4 Stress distribution and osteointegration

Frost's hypothesis states that bone cells respond to local deformation of the bone produced by mechanical stress (Frost, 1987). Bone adapts its strength in response to the applied load. If the strain in the bone surrounding an oral implant is in the “mild overload” range (1,500–3,000 microstrain), apposition of bone seems to be the biological response. On the other hand, strain beyond this range will alter the phase of cell activity leading to osteoclasis result in fatigue fracture and bone resorption (Isidor, 2006).

Naturally, it is difficult, clinically, to quantify the magnitude and direction of the forces occurring in the surrounding bone during implant insertion (Guan et al., 2011) and how the cells respond to the stress immediately after implantation. However, clinical studies have used the resonance frequency analysis technique on various implant designs to determine implant stability, reflecting the peri‐implant bone status. A previous study of the relationship between the stability of PW Plus® dental implants and two biological markers during the healing period has shown that the mean implant stability quotient (ISQ) values decreased between 1 and 3 weeks after implantation. After 4 weeks, the ISQ values recovered to the initial values (Tirachaimongkol et al., 2016). These results are similar to those of Rabel et al., who showed high‐mean ISQ values immediately after implantation of Camlog® dental implants, and again, 3 months later (Table 3; Rabel, Köhler, & Schmidt‐Westhausen, 2007). The results of these two studies can be applied to the bone remodeling process after implantation. Therefore, in this finite element study, the range of the stress magnitude in the surrounding bone may improve the bone‐healing process. However, the directional biological responses of the bone cells resulting from various stress magnitudes after implantation require further study.

**Table 3:** Means of the resonance frequency analysis immediately after insertion and after 3 months

	Mean RFA (ISQ; immediately post‐op)	Mean RFA (ISQ; 3 months post‐op)
PW Plus® (Tirachaimongkol et al., 2016)	77	79.6
Camlog® (Rabel et al., 2007)	64.4	67.3

### 4.5 Limitations

In this study, the bone structures in the models were all assumed to be homogeneous, isotropic, and linearly elastic. The properties of the cortical bone of the mandible were transversely isotropic and inhomogeneous; however, for the sake of simplification, this was ignored in the modelling of this research. These are inevitable limitations of this study.

This simulation model used a quarter segment of the bone instead of a 360° area surrounding the bone to present the stress profile. Due to the reduction in the computational cost of the simulation, the quarter model was efficient to represent the stress magnitude during the implant insertion. In addition, the reduction of the bone contact surface area allowed for the implant to move without severe element distortions, which could have led to aborting the simulation.

Two factors affecting the simulation were implant material and surface roughness. Optimum implant material properties lead to increased bone regeneration and early stabilization of the dental implant system (Shamami, Karimi, Beigzadeh, Haghpanahi, & Navidbakhsh, 2014; Shirazi, Ayatollahi, Karimi, & Navidbakhsh, 2016). Increased surface roughness increases the friction coefficient between the material surfaces (Dos Santos, Elias, & Cavalcanti Lima, 2011). In this study, the implant details of the surface roughness were ignored because the study focused only on the effect of thread design.

Although FEA has been extensively used to predict the biomechanical performance of various dental implant designs, as well as the effect of clinical factors on the success of implantation, it is still necessary to further evaluate and understand the correlation of many variables in the long‐term success of dental implants to improve outcomes. These variables include a more precise simulation process with realistic material properties and geometry, variation of the bone porosity, design parameters, roughness of the implant surface, analysis techniques, and variation of the insertion conditions.

## 5 CONCLUSIONS

Within the limitations of the study, it can be concluded that in the same boundary condition, different implant designs affect different stress generation patterns during implant insertion. Implant surface contact area is a major factor impacting the stress characteristics during implantation. A range of stress magnitude, generated in the surrounding bone during implant insertion, may influence bone healing around dental implants and final implant stability.

## FUNDING INFORMATION

This study was supported by Chiang Mai University research fund.

## CONFLICT OF INTEREST

There were no conflict of interests in connection with this study.

## Supporting information

Video S1. Supporting information

Video S2. Supporting information

Video S3. Supporting information
